# Preface

**DOI:** 10.1098/rstb.2008.0012

**Published:** 2008-02-11

**Authors:** Yadvinder Malhi, Timmons Roberts, Richard A Betts

We stand at a time of crisis and opportunity for the tropical forest regions of the world that are home to half of all global biodiversity. On one hand, the pressures of deforestation driven by the infrastructure expansion in tropical forest frontiers and accelerating global demand for soya, beef, palm oil, timber and biofuels produce unprecedented pressure for rapid and accelerated deforestation and degradation. For the Amazon region in particular, this pressure is tightly coupled with increased probability of drought and fire occurrences driven by both local deforestation and global climate change. On the other hand, there is a new appreciation of the role that tropical forest preservation can play as a component of a strategy to mitigate global climate change. The UN Climate Change Conference in Bali (December 2007) has agreed to include payments for Reduced Emissions from Deforestation and Degradation within the framework of the Kyoto Protocol, thus potentially facilitating an unprecedented increase in the amount of funds and political will available for tropical forest conservation. At the same meeting, Brazil announced that it intended to bring a complete halt to deforestation in its Amazonian region within a decade. However, the effective implementation of such ambitions raises many challenges to our knowledge, such as (i) a scientific understanding of the climatic threat and its synergy with land-use change, (ii) an ecological understanding of the resilience or vulnerability of Amazonian biomes, (iii) a social, political and economic understanding of the dynamics and drivers of deforestation and degradation at forest frontiers, and (iv) a political and historical understanding of why policy measures have historically failed to control deforestation in the Amazon frontier.

This raises a challenge to all scientists interested in the future of tropical forests, and such a complex environmental problem requires an interdisciplinary analysis. This volume attempts to provide such an analysis for the future of the Amazon. It arises from an international workshop held at Oriel College, Oxford, UK, in March 2007, and brings together 25 original papers on some of the latest research about the drivers, processes and impacts of environmental changes in the Amazon, and potential new policy responses. We would like to thank the James Martin 21st Century School, the Environmental Change Institute, Oriel College and the Centre for Brazilian Studies (all at the University of Oxford) for generously supporting the workshop, and the Jackson Foundation, the College of William and Mary and the Met Office Hadley Centre for supporting the time that we as organizers spent in implementing both the workshop and this special issue. We thank the UK Department for Environment, Food and Rural Affairs (contract GA01101) for providing funding to enable this special issue to be freely available online. We would like to extend particular thanks to Diana Liverman for her warm support of the initial concept, to Jane Applegarth and Deborah Strickland for their extensive administrative support, and to James Joseph at the Royal Society for patiently and cheerfully seeing this thematic issue through to completion. Podcasts and slides of the original conference proceedings are available at http://www.eci.ox.ac.uk/news/events/070320presentations.php.

The challenge of maintaining the ecosystem services of Amazonia in the face of deforestation pressures and climate change will require interdisciplinary research and analyses that span the climatological, ecological, social, political and economic sciences, and interface effectively with regional and international policy. We hope that this volume makes a useful contribution to this analysis.

